# Pathprinting: An integrative approach to understand the functional basis of disease

**DOI:** 10.1186/gm472

**Published:** 2013-07-26

**Authors:** Gabriel M Altschuler, Oliver Hofmann, Irina Kalatskaya, Rebecca Payne, Shannan J Ho Sui, Uma Saxena, Andrei V Krivtsov, Scott A Armstrong, Tianxi Cai, Lincoln Stein, Winston A Hide

**Affiliations:** 1 Department of Biostatistics, Harvard School of Public Health, 655 Huntington Avenue, Boston, MA 02115, USA; 2 Bioinformatics Core, Harvard School of Public Health, 655 Huntington Avenue, Boston, MA 02115, USA; 3 Ontario Institute for Cancer Research, Department of Informatics and Bio-computing, MaRS Centre, South Tower, 101 College Street, Toronto, ON, M5G 0A3, Canada; 4 Division of Hematology/Oncology, Boston Children's Hospital, Harvard Medical School, 300 Longwood Avenue, Boston, MA 02115, USA; 5 Harvard Stem Cell Institute, 1350 Massachusetts Ave, Cambridge, MA 02138

## Abstract

New strategies to combat complex human disease require systems approaches to biology that integrate experiments from cell lines, primary tissues and model organisms. We have developed Pathprint, a functional approach that compares gene expression profiles in a set of pathways, networks and transcriptionally regulated targets. It can be applied universally to gene expression profiles across species. Integration of large-scale profiling methods and curation of the public repository overcomes platform, species and batch effects to yield a standard measure of functional distance between experiments. We show that pathprints combine mouse and human blood developmental lineage, and can be used to identify new prognostic indicators in acute myeloid leukemia. The code and resources are available at http://compbio.sph.harvard.edu/hidelab/pathprint

## Background

Complex human diseases arise from perturbations of the cellular system [1]. Defining these changes from a systems biology perspective provides the opportunity to relate the function of genes, pathways, and processes. The ability to compare experiments across model organisms and humans directly influences our capacity to determine the basis of disease [2-4], and the importance of cross-species data analysis has been well illustrated: human disease genes have been identified by large-scale meta-analysis of conserved human-mouse co-expression [5], gene-based cross-species distance metrics have highlighted diseases that activate similar human and mouse pathways [6], and oncogenetic expression signatures have been prioritized by comparing human cancer and mouse model expression profiles [7-9]. Gene expression provides the most extensive resource to profile functional changes, and the opportunity for large-scale meta-analyses has been made possible by the development of public data repositories such as the National Center for Biotechnology Information Gene Expression Omnibus (GEO) [10] and the European Bioinformatics Institute ArrayExpress [11]. Cross-study analysis and integration is an area of extremely active research; however, most gene-based approaches are confounded by the challenge of comparing gene activity between different platforms and species. Consistent and scalable methods for combining these data are now required so that researchers can perform comprehensive integration of existing knowledge with new experiments, identify consistent signals, compare heterogeneous data, and validate hypotheses.

Methods for cross-study integration of gene expression data have tended to focus on differential expression in well-matched control and experimental samples [12], because approaches based on correlation or absolute profiles [13] are dominated by laboratory and platform variability in cross-study analyses [14]. The ability to leverage public data to address platform-effects has been demonstrated most recently by the Gene Expression Barcode (GEB) and Gene Expression Commons, both of which define absolute gene expression scores based on a background distribution [15,16]. However, by virtue of their reliance on gene level comparisons, these compelling simplifying approaches are restricted to selected platforms, and so do not address global comparison of biological function across experiments and species.

We sought to develop a new function-based approach for comparing profiles, which can truly scale across the diversity of available experiments, platforms, and species. Expression of biological functions across batches and divergent expression platforms shows higher concordance than across genes [17], and assigning genes to pathways [18-20] or ontologies [21] is effective for revealing phenotype associations [22-25], performing cross-platform integration [14], and specifying disease subgroups [26]. To this end, we have developed Pathprint, a global pathway activation map spanning 6 species and 31 array technologies, which represents expression profiles as a ternary score (underexpressed (-1), intermediately expressed (0), or overexpressed (+1)) in a set of 633 pathways, networks, and transcriptionally regulated targets. The method leverages a static background built from public data repositories, integrating pathway annotation and prediction with large-scale profiling.

Pathprint provides both a quantitative definition of cellular phenotype and a functional distance between all experiments, based on their global pathway activity. It presents a significant methodological advance over single-study, relative enrichment methods such as Gene Set Enrichment Analysis (GSEA) [27] and existing gene-based methods for comparison between platforms and species. Pathprinting provides a robust framework for large-scale meta-analyses of clinical data, and allows phylogenetic reconstruction of developmental lineages from a functional perspective. We demonstrate the use of pathprinting for retrieval of functionally matched samples from cross-platform expression databases, reconstruction of the blood developmental lineage across species, and integration of data from mouse experiments, human samples, and clinical studies to develop new prognostic indicators and drug targets in acute myeloid leukemia (AML).

## Methods

**Figure 1:**
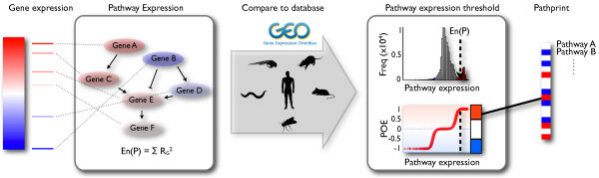
**The Pathprint pipeline**. Rank-normalized gene expression is mapped to pathway expression. A distribution of expression scores across the Gene Expression Omnibus (GEO is used to produce a probability of expression (POE) for each pathway. A pathprint vector is derived by transformation of the signed POE distribution into a ternary score, representing pathway activity as significantly underexpressed (-1), intermediately expressed (0), or overexpressed (+1).

The pipeline to create a pathprint of an array is shown in Figure 1. A score of 0 in the final pathprint vector represents pathway expression at a similar level to the majority of arrays of the same platform in the GEO database, while scores of 1 and -1 reflect significantly high and low expression respectively. Below we describe the individual steps used to construct the method.

### Expression data for building pathway background distributions

A list of arrays from 31 of the most highly represented one-channel gene expression platforms in GEO that profiled *Homo sapiens*, *Mus musculus*, *Rattus norvegicus*, *Danio rerio*, *Drosophila melanogaster*, and *Caenorhabditis elegans *was compiled (see Additional file 1) and the normalized expression tables retrieved. All normalization methods were accepted. After discarding incomplete records, this list contained 176,971 arrays. It was necessary to restrict the platform coverage to one-channel arrays, because two-channel arrays provide the relative expression of genes between test and control samples, hindering direct comparison of the test sample between experiments when the control sample differs. The expression data were mapped to Entrez Gene identifications (IDs) using systematically updated annotations from AILUN (Array Information Library Universal Navigator) [28]. Multiple probes were merged to unique Entrez Gene IDs by taking the mean probe set intensity. It should be noted that although the mean expression level will produce stable gene expression values, it also 'averages out' the effects of alternative promoter usage and splice variants. Tissue-specific splicing has been recognized as an important factor in defining cellular function [29]; however, at the present time, there are insufficient data to allow consistent mapping of individual splice variants to pathways.

### Pathway databases

Canonical pathway gene sets were compiled from Reactome [18], Wikipathways [20], and KEGG (Kyoto Encyclopedia of Genes and Genomes) [19], which were chosen because they include pathways relating to metabolism, signaling, cellular processes, and disease. For the major signaling pathways, experimentally derived transcriptionally upregulated and downregulated gene sets were obtained from Netpath [30]. The pathways provide structured relationships between genes, unlike ontologies such as the Gene Ontology (GO) database [21] that define relationships between but not within terms.

### Static modules

Pathprint is built to leverage expertly curated biological knowledge found in canonical pathway databases within a systematic framework. This approach provides a consistent biological annotation of datasets in terms that are well understood by the community. However, a uniquely pathway-centric approach would introduce an inherent curation bias towards well-studied genes and processes. Therefore, we have supplemented the curated pathways with non-curated sources of interactions by including highly connected modules from a functional-interaction network, termed 'static modules.' This functional-interaction network was constructed by extending curated pathways with non-curated sources of information, including protein-protein interactions, gene co-expression, protein domain interaction, GO annotations and text-mined protein interactions. The final functional-interaction network contains 181,706 interactions between 9,452 genes [31], representing close to 50% of the total human proteome. A Markov cluster algorithm was applied to decompose the network, yielding 144 closely related functional-interaction clusters or 'static modules', ranging from 10 to 743 nodes. Each cluster was named according to the member gene with the highest interaction degree. The modules cover 6,458 genes, 1,542 of which are not represented in any of the pathway databases. These static modules offer the opportunity to examine the activity of less studied or annotated biological processes, and also to compare their activity with that of the canonical pathways. To provide biological context for the static modules, the top GO terms associated with all the pathways have been compiled (see Additional file 2).

### Compiling cross-species gene sets

*M. musculus*, *R. norvegicus*, *D. rerio*, *D. melanogaster*, and *C. elegans *gene sets were inferred using homology based on the HomoloGene database [32]. HomoloGene uses pairwise gene comparison combined with a guide tree and gene neighborhood conservation. HomoloGene was selected because, compared with alternative inference methods, it provides a better functional proxy and higher specificity for the resolution of shared cellular ontogeny, albeit with lower overall coverage [33].

### Summary of the pathprint gene sets

All of the modules and pathways were converted to flat gene sets, so intra-pathway gene-level interaction data were not used. Combined, the canonical pathways, downstream targets, and static modules totaled 633 human gene sets. The gene membership of these sets is described in Table 1, within the R package Pathprint, and on the Pathprint website (for the number of genes overlapping between each of the data sources see Additional file 3). Specific pathway sub-sub-sets may also be used in individual analyses.

**Table 1:** Summary of gene sets used in Pathprint

	Pathways, n	Mean size, n	Median size, n	Minimum size, n	Maximum size, n	Total genes, n
Reactome	53	154	108	11	932	4,874
Wikipathways	173	50	33	6	260	3,918
Netpath	36	170	83	8	816	3,811
KEGG	227	76	55	6	1,138	5,990
Static modules	144	45	21	9	733	6,458
All	633	74	41	6	1,138	10,903

Abbreviations: KEGG, Kyoto Encyclopedia of Genes and Genomes.

#### Calculating pathway expression

Genes were ranked by expression level, from 1 (low expression) to *T *(high expression), where *T *is the total number of genes in the array. For a pathway, *P*, of size *k*, represented in an array by genes *G_1_, G_2_...G_n_*, the pathway expression score, *En(P)*, is defined by the mean squared rank

$$En\left(P\right)=\frac{1}{n}\times \overset{n}{\underset{i=1}{\sum }}R_{i}^{2},$$

where *R_i _*is the rank of gene *G_i _*in a pathway containing *n *genes. Rank normalizations provide robust summary statistics to calculate pathway expression scores [6,13] that can be applied across all technologies, and does not depend on the dynamic range of an array. The mean squared rank was chosen based on a survey of statistical approaches for gene set analysis [34], and out-performed other summary statistics in a series of classification benchmarks based on tissue-specific pathway expression (see benchmarking section below).

#### Normalization and probability of expression

When comparing gene-set expression scores between experiments, it is essential to assess the expression against a suitable null hypothesis [35]. In this case, comparison of the expression of a gene set in one array with its expression in all other arrays, that is, sample permutation, is required to account for the internal gene expression correlation structure within gene sets, which is expected to be particularly high within pathways [36]. For each gene set, the expression score was normalized against a background built using all arrays of the same platform type. To our knowledge, this is the first study comparing database-wide gene set expression, and the expected distribution scores are not known. We adopted a similar approach to the GEB [15], which estimates which genes are expressed and which are unexpressed in data from single microarrays. The GEB converts gene expression levels to binary scores based on a static background distribution built from public expression data for three distinct platforms. In this study, we constructed static pathway expression background distributions for each pathway across 31 platforms in GEO [10], and then fitted each of these distributions to a two-component uniform-normal mixture model [37]. The normal component represents the core distribution of pathway expression scores for a particular pathway, that is, not significantly high or low expression. The uniform component represents outlying pathway expression due to significantly high or low expression. A signed probability of expression (POE), representing the probability that a pathway expression score belongs to the uniform component of the fitted mixture model, can be calculated. We took advantage of the increase in computation speed afforded by the expectation-maximization implementation of POE in the R package metaArray [38].

### Application of a ternary threshold

High/low thresholds [15], or filters with weight vectors approaching the thresholding limit [6], operate as an effective noise filter to remove uninformative signal variation. POE values were converted to a ternary score by the transformation:

$$\begin{array}{c} F_{i}=1:T\leq POE_{i}\\ F_{i}=0:-T<POE_{i}<T\\ F_{i}=-1:POE_{i}\leq -T, \end{array}$$

where *POE_i _*(*i *= 1, 2... *n*) represents the POE for gene set *i*, *T *is the threshold and *F_i _*are components of the pathprint vector. Selection of the threshold, *T*, is of vital importance, as this directly modulates the sensitivity and specificity at which gene sets are scored as significant. Large values of *T *(high stringency) is appropriate for gene expression [15], while small values of T (low stringency) increase the weighting of subtle differences in expression, and may be required to discriminate arrays at the pathway level, where the coordinated effects of multiple genes are under consideration. The threshold was optimized by combining multiple benchmarks (see below). Thresholding improves sample clustering (see below), provides a read-out for sample annotation, and simplifies quantification of sample relationships.

### Constructing consensus pathprints

To summarize the activity of a group of pathprints, we defined the consensus score for each pathway as

$$\begin{array}{c} C_{i}=1:\mu_{i}>t\\ C_{i}=-1:\mu_{i}>-t, \end{array}$$

where *μ_i _*is the mean score for pathway i across the group of pathprints, and *t *is a consensus threshold value. The consensus pathprint is the vector constructed by calculating the consensus score for each pathway, representing the consistently significantly expressed pathways across the group. The rationale behind introducing a threshold is to associate a set of pathways with a phenotype, and so provide a discrete functional representation of a cell type based on a collection of pathprints.

### Defining distance between pathprints

A functional distance between experiments is defined as the distance between two pathprint vectors. We defined the distance by the Manhattan distance, providing a simple read-out for the number of pathway scores differing between two samples. We defined the distance from a consensus pathprint to any other pathprint by the Manhattan distance between the subset of the pathprint vectors that contain only the pathways for which the consensus pathprint is non-zero. This ensures that only differences in the consistently expressed pathways that make up the consensus pathprint are considered.

### Optimizing threshold value

The threshold value was optimized using cross-platform, cross-species gene expression data from a panel of human and mouse tissue samples [15] and an independent dataset profiling brain sub-regions in human, mouse, and rat [39].

Four approaches were used to determine the optimum threshold.

#### 1) Cross-validation

The datasets were divided into five sub-sets of equal, or approximately equal, size. One of the sub-sets (the test set) was omitted, and mean pathprints were calculated for each tissue from the remaining samples (the training set). Next, the samples in the test set were assigned to the tissue with the closest mean tissue pathprint in the training set by Euclidean or Manhattan distance (both yielded similar results). An error rate was calculated by comparing these assignments with the known annotations. This was repeated, omitting each of the sub-sets in turn, to obtain a mean error rate. The cross-validation procedure was performed 10 times for each threshold value to estimate the mean and standard deviation (SD) of the error rate. The SD was small relative to the change in mean error rate over the thresholds, and so this number of repetitions was deemed sufficient (see Additional file 4). The procedure was also performed as a 'leave-one-out' cross-validation, equivalent to dividing the data into a number of sub-sets equal to the same number of samples, with similar results.

#### 2) Cluster validity (intra-tissue versus. inter-tissue distance and principal components analysis)

Cluster validity was determined by the ratio of the intra- to inter-tissue variance, where variance was defined as sum of the squared Euclidean distance between each sample and the mean pathprint for each tissue. A lower ratio indicates tighter clustering within tissues and/or better separation of the tissue type clusters. The clusters formed by pathprints had an intra-cluster/inter-cluster distance ratio of 0.63, compared with 1.26 for GEB and 0.92 for Spearman correlation (see Additional file 4).

#### 3) Retrieval: precision recall of cross-species tissue data

**Figure 2:**
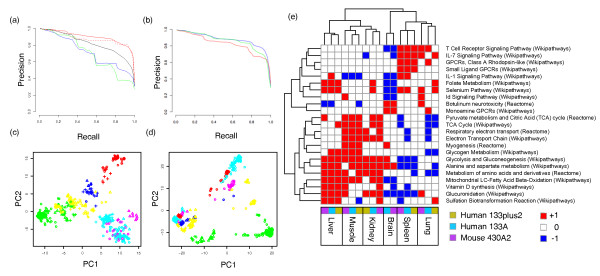
**Cross-species integration**. **(a) **Precision recall within the tissue training dataset for the pathprint (red indicates mean average precision (MAP) = 0.90), unthresholded POE (dashed; MAP =, 0.88), random gene sets (black, MAP = 0.83), Gene Expression Barcode (blue, MAP = 0.73), Spearman gene expression correlation (green, MAP = 0.71). **(b) **Comparison of distance metrics; precision-recall curves for aggregated mouse to human tissue data based on a thresholded pathprint build produced using Euclidean (blue), Manhattan (green), and Mahalanobis (red) distances. **(a,b) **Tissue-dominated versus platform/species-dominated clustering showing plots of the two most significant principal components (PCs) for **(c) **the pathprint and **(d) **the Gene Expression Barcode (red, brain; yellow, kidney; green, liver; light blue, lung; dark blue, muscle; pink, spleen; circles, Mouse 430A2; diamonds, Human 133plus2; crosses Human 133A). **(e) **Functional classification of tissues and blood cell types. Hierarchical clustering of consensus pathprints for human and mouse tissues on three platforms based on the Wikipathway and Reactome pathways that significantly contributed to clustering. Colors indicate scores: red, 1; white, 0; and blue, -1).

The combined human and mouse dataset was ranked by distance from each sample (Manhattan, Euclidean, or Spearman correlation). These ordered retrieval lists were used to calculate average interpolated precision-recall curves at a range of threshold values. Decreasing the stringency of the threshold initially improved performance, but at thresholds of less than 0.001, the difference became less significant, summarized by the plot of mean average precision (see Additional file 4). Pathprinting improves the performance of tissue retrieval across species compared with gene expression measurements (both GEB and Spearman correlation) and results obtained with randomly constructed gene sets (Figure 2). Pathway expression scores based on the mean squared rank out-performed the mean rank, as assessed by precision-recall curves for the tissue-species data. In addition, an identical analysis pipeline was also constructed using the GSEA algorithm, as applied to single samples [25], as the initial step, in place of the mean squared rank. It was found that the enrichment scores were highly correlated, and yielded no significant improvements in precision or recall. There was also a much greater computational burden associated with running GSEA on 180,000 arrays compared with using the mean squared rank on the same number of assays.

#### 4) Comparison with randomly constructed gene sets

A pathprint based on 'random' gene sets was constructed to test whether the 'expert' knowledge contained within the pathways and modules contributed to the success of the pathprint, over and above the effect of simply reducing the dimensionality of the data. These random gene sets contained genes sampled without replacement from the genes used in the original pathways, and retained the size distribution of the original pathway list. The performance of the precision-recall curves for pathprint based on random gene sets (Figure 2; see Additional file 4) were inferior to pathprint, and this was especially pronounced at stringent thresholds. At less stringent thresholds, the difference between the curves was smaller, implying that both the reduction in data dimensionality and the integration of biological knowledge contribute to the effectiveness of pathprint.

A threshold value of 0.001 was chosen on the basis that it performed optimally across the majority of the performance measures. It is interesting to note that a highly stringent threshold, approximately 0.9, did not perform well in cross-validation but yielded good results for the precision-recall and cluster-validity tests, and produced the greatest difference in performance compared with the equivalently thresholded random gene sets. These results show that moderate pathway expression levels best characterize samples, but the most highly expressed pathway expression scores are also informative. Further work is required to determine whether combining more than one thresholding regimen would be beneficial.

### Phenotype matching using the GEO database

Any set of arrays, such as tissue-specific arrays, can be used as a 'seed' to construct a consensus pathprint profile representing the commonly expressed functions of the set (Figure 2). The distance of every array in the GEO pathprint collection can then be measured to produce a table of GEO samples, ordered on the basis of their phenotypic similarity to the seed set, that is, a ranked list of retrieved samples (see Additional file 5).

### Distribution of distances

In considering the distribution of distances from a consensus pathprint, a major problem is how to assign a measure of significance. This is particularly important if it is necessary to impose a cut-off point at which to evaluate retrieved results. Calculating significance based on the distribution of pathprint scores across the full GEO database is complicated because 1) each pathway has a different distribution of ternary scores, and 2) the pathways scores are known to be correlated. An alternative strategy is to use the distribution of the database to define a background distribution, based on the following assumptions: first, that there are two distinct populations, namely, a small number of closely matched and a large number of non-matched samples; and second, that the distances of the non-matched samples are normally distributed. The estimated distribution of the non-matched samples is derived from the interquartile range of the full distribution. The significance with which an array is matched with a pathprint, or with a consensus pathprint, is then calculated using the *P*-value, based on the normal distribution function based on this estimated distribution. This approach is clearly an oversimplification, and a more complete significance model will form the basis of further study. We expect a large number of the samples contained in GEO to be disease-related, representative of a research focus bias inherent in the scientific literature, and so we are aware that the underlying distribution could be multimodal, owing to perturbed transcriptional programs and copy-number variations associated with disease, specifically cancer cell types. The correlation between this estimated *P*-value and the precision for each of the six tissue samples is shown (see Additional file 5).

### Phylogenetic analysis

Pathprints corresponding to hematopoietic gene expression datasets GSE24759 [40] and GSE6506 [41] were calculated using the pathprint pipeline. A consensus pathprint was constructed for each of cell types using an arbitrarily selected threshold of 0.75. Phylogenetic analysis was performed using the R package Phangorn [42]. Optimized parsimony and (non-parametric) bootstrapped trees were found by nearest neighbor interchange with a cost matrix based on the difference between pathprint scores.

### Self renewal-associated signature and survival analysis

Gene expression data for leukemia stem cells, normal stem cells, and progenitor cells in mouse and human were obtained from the GEO database (GSE24006 and GSE3722). Pathprints were calculated for each sample using the Pathprint package in R. Pathways shared by leukemic and normal stem cells that are differentially expressed in progenitor cells were identified for the human and mouse datasets. The self renewal-associated signature (SRAS) was defined as the set of pathways common to the human and mouse signatures. Gene expression arrays and the associated survival data were obtained from GEO for four clinical studies of AML (GSE10358, GSE12417, GSE1159, and GSE14468). Pathprints were calculated for each sample in these datasets. Survival plots and associated *P-*values were derived using the Kaplan-Meier method by stratifying patient samples into two groups by the sum of their pathprint scores across the SRAS pathways. For each dataset, the approach was repeated 1,000 times using random permutations of the pathprint pathways with the same number of member pathways as the SRAS set to produce a background distribution of *P*-values against which to compare the SRAS result.

### Code and Pathprint R package

The code and data to process gene expression arrays to pathprints have been compiled into the R package Pathprint. Pathprints have also been pre-calculated for approximately 180,000 gene expression profiles from the GEO repository and included in the R package, along with their associated metadata, in order to create a searchable cross-platform matrix covering 31 platforms and 6 species (see Additional file 1). Future versions of Pathprint will extend the acquisition pipeline to encompass the remaining platforms and incorporate data from other repositories. The package and the complete R code (as Sweave documents) required to reproduce the analysis and figures contained within this manuscript are available online [43].

## Results and Discussion

The ability of pathprints to classify cross-platform and species data was tested on a series of tissue-specific datasets, and compared with the GEB [15], gene expression correlation, and a pathprint based on random gene sets (Figure 2; see Additional file 4). In each test, pathprints improved sample classification, and clustered tissues together across platform and species. The biological and technical variation across pathprints in the tissue-specific dataset was investigated by principal components analysis (Figure 2c). The first two principal components separated most tissue types, irrespective of their originating platform and species, with some convolution of the lung and spleen samples. Notably, a corresponding plot produced from GEB data clustered samples first by platform and then tissue type (Figure 2d).

A high degree of overlap in gene membership is introduced when combining multiple pathway databases. Overlapping gene membership can be due to redundancy in the pathway sets, for example different views of the Wnt pathway in the Reactome, Wikipathway, and KEGG databases, or due to a close biological relationship between pathways and so sharing of a subset of their genes, such as 'G1 to S cell cycle control' and 'DNA replication'. Overlapping genes will result in correlation between the gene expression scores of these pathways. In addition to the correlation due to overlapping genes, it is well recognized that pathways do not function as discrete elements, but rather are organized into cascades and co-regulatory networks. We did not attempt to make a quantitative definition of the second source of correlation, but we did test the effect of correcting for overlapping genes by incorporating a pathway covariance matrix to adjust the contribution of each gene set using the Mahalanobis distance. The covariance matrix was calculated using pathway expression scores from 10,000 randomly permuted expression profiles to providing a measure of the covariance due to the gene-member overlap, without the additional complication of gene-gene expression correlations. In the benchmark tests, the Mahalanobis distance did not improve performance over the simpler Euclidean and Manhattan distances (Figure 2b), thus, all pathways, irrespective of size and including overlapping gene sets, were retained in the pathprint. No additional correction was made, as we wished to maximize the utility of the pathprint as a source of annotation of samples and for sample clustering and organization. Plans to include feature selection of gene sets that contribute the most toward performance, for example by non-negative matrix factorization, are the subject of ongoing algorithmic development.

We will now outline a series of case studies demonstrating major applications of pathprinting, focusing on integrating data from human and mouse.

### Tissue-specific pathway profiles

The consensus pathprints derived from the tissue-specific datasets described above define consistent functional identities for each tissue; for example, skeletal muscle expresses myogenesis, liver and kidney express metabolic pathways, and brain expresses neuroactive ligand receptors (Figure 2e). To validate these tissue-specific pathway combinations, the full GEO matrix of pathprints, approximately 180,000 samples, were ranked based on pathprint distance from each tissue profile. Originating tissue types were assigned for each GEO sample using the metadata in the database, allowing validation of the matched samples and the construction of precision-recall curves for each tissue. The results showed remarkable specificity (Table 2; see Additional file 5): the 50 human and mouse brain Affymetrix arrays used to build a brain profile retrieved approximately 8,500 brain samples at 95% precision, spanning 4 species (human, mouse, rat, and zebrafish) and 25 different platforms (from Affymetrix, Illumina, and ABI). For 5 of 6 tissues, over 1,000 correctly matched arrays were retrieved at 95% precision. Although performance was noticeably worse for spleen, a high proportion of spleen mass is blood, and therefore blood samples, predominantly leukocytes, ranked highly in the retrieval list, lowering the observed precision. We tested the ability of the brain and liver consensus pathprints of mouse and human to retrieve samples from each of the other species covered by the pathprint; rat, zebrafish, fruit-fly, and nematode. The top matches for the brain consensus were all brain samples for rat and zebrafish, head samples for fruit-fly, and a more heterogeneous set that included neuron samples for nematode. The top samples retrieved by the liver consensus were liver in rat and zebrafish, and whole samples for nematode and fruit-fly (see Additional file 6).

**Table 2:** Pathprint-based retrieval of data from the Gene Expression Omnibus (GEO); arrays retrieved from GEO from consensus tissue pathprints at 95% precision

	Seed arrays, n	Correct retrievals, n	Platforms, n	Species, n
Brain	50	8,691	25	4
Kidney	81	1,156	14	3
Liver	196	4,797	22	4
Lung	142	1,735	13	3
Skeletal muscle	29	2,919	18	3
Spleen	33	179	5	2

### Development of a pluripotent pathprint

The study and characterization of embryonic stem cells (ESCs) is dominated by subjective choices of selection markers. ESCs express consistent transcriptional profiles that provide benchmarks for pluripotency [44]; however, to date, it has not been possible to consistently assess ESC signatures across all available data and platforms, and it is becoming increasingly important to provide biologically interpretable functional signatures that are robust across a range of experimental origins. An ESC pathprint was derived from 127 human and mouse samples (see Additional file 7) that includes high expression of known ESC-related functions such as DNA repair, one-carbon metabolism [45], and a network centered on *SUMO1*, the ubiquitin-related modifier thought to target and stabilize Oct4 [46]. The profile is a consistent indicator of pluripotency; 90% of the 1,000 closest pathprint-matched samples in GEO are ESCs and induced pluripotent stem cells (iPSCs) from 140 different human and mouse studies and 13 platforms (see Additional file 8; see Additional file 9). The non-ESC/iPSC samples retrieved were cancer cell lines known to express ESC pathways, consistent with the concept that pathways required for stem cell specification play fundamental roles in tissue regeneration and cancer. Systematically profiling stem cells using pathprints to integrate data from mouse models, human primary tissue, and clinical studies will resolve the contributions of these stem cell pathways to developing and aberrant systems and reveal pathways of clinical relevance.

### Integration of the human and mouse hematopoietic lineage

**Figure 3:**
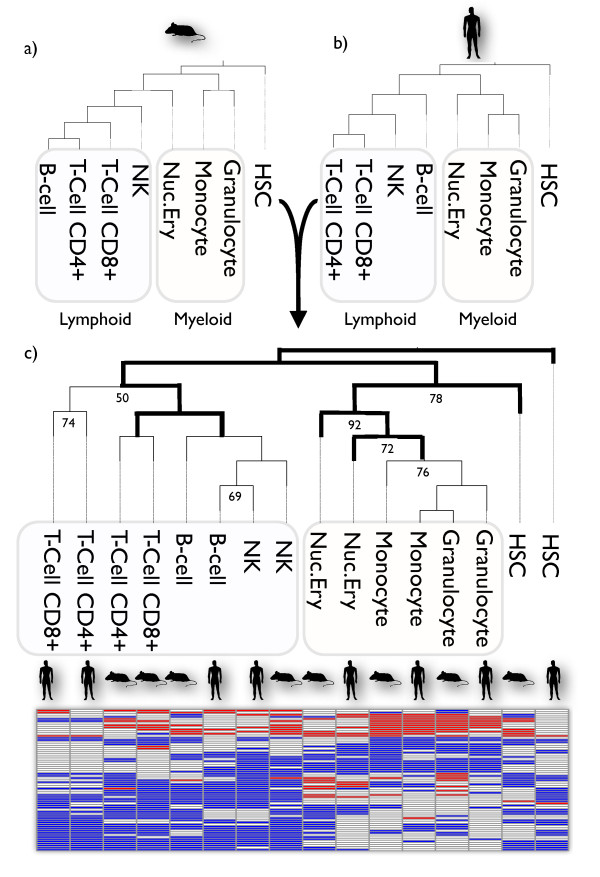
**Functional classification of blood cell types**. (a) Maximum-parsimony phylogenetic reconstruction of the hematopoietic lineage using pathprints calculated from **(a) **human [40] and **(b) **mouse [41] gene expression experiments. **(c) **Combined human-mouse tree based on shared informative pathways that resolve trees (a) and (b) and the pathway heat-map. The myeloid (yellow) and lymphoid (purple) branches are indicated, and dark branches represent agreement with the canonical lineage. See Additional file 10 for pathway annotations.

Mapping cellular lineages has traditionally relied on direct observation, or on endogenous or genetically engineered markers. Defining cell types using a combination of markers is not always possible, and often the link between marker and cellular function is not understood. Hematopoietic differentiation has been analyzed in the context of the canonical view of blood lineage using gene expression profiles of surface marker-purified cell populations in human [40] and mouse [41]. Pathprinting allows a novel pathway-based phylogenetic approach for an unsupervised definition of this lineage by maximum-parsimony reconstruction using the discrete pathprint states. The reconstruction recapitulates the known lineage ontogeny, and allows integration of human and mouse data, using the common informative pathways (Figure 3; see Additional file 10). The phylogeny resolves the major myeloid and lymphoid branches, independent of species. Species-specific contributions overcome some cell-type groupings, but this is unsurprising, because marker selection and immune presentation differ between the experiments. A comprehensive survey of mouse immune-cell gene expression is in progress [47]. As these and further data become available, pathprints will allow integration with the existing human and mouse ontogenies, identifying functional differences, and resolving problems of data availability and incomplete lineage coverage.

### Self-renewal pathways in acute myeloid leukemia

**Figure 4:**
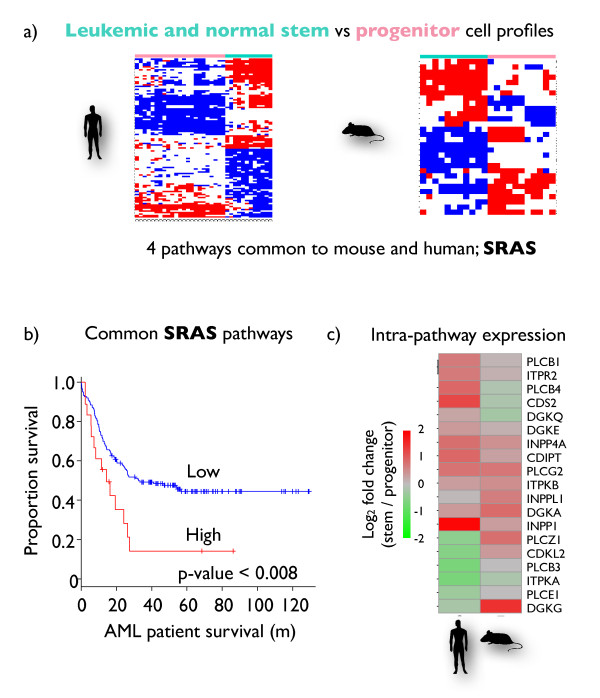
**Clinically important self renewal-associated signature (SRAS) in acute myeloid leukemia (AML)**. **(a) **Pathways differentially expressed in stem and non-stem cell profiles in leukemic and normal samples were found in human and mouse experiments. Four common SRAS pathways were identified. **(b) **The SRAS pathprint scores of patients with AML were significantly associated with survival. **(c) **A single pathway of interest is highlighted, the overall PGCL2 (α 2u globulin) module is upregulated in normal and cancer stem cells but individual genes differ between species. This pathway is strongly associated with survival (see Additional file 13).

Well-characterized mouse models of AML (AML) have been used to explore the molecular basis for the stem cell-like behavior of sub-populations of leukemia cells [48]. An SRAS that is activated in both hematopoietic stem cells and leukemia initiating cells has been identified. An analogous study of human AML has identified a clinically relevant stem cell-associated signature expressed in human normal hematopoietic and leukemia stem cells [49]. There are only four genes common to the published human and mouse signatures, and the extent to which the mouse model functionally recapitulates the human system is unknown. A pathprint analysis systematically extracted and compared the pathways defining stem phenotypes in each of these studies, identifying four common human and mouse stem cell-associated pathways. The common pathways are translation factors and class B secretin-like G protein-coupled receptors (GPCRs) from Wikipathways, and static modules centered on *1-phosphatidylinositol-4,5-bisphosphate phosphodiesterase gamma-2 (PLCG2) *and *RAS-related nuclear protein *(*RAN) *(Figure 4a). There is no overlap between these pathways at the gene level. The combinatorial clinical relevance of these pathways was tested by calculating pathprints for four independent clinical studies of gene expression in patients with AML [50-53]. The patient samples were grouped into high-expression and low-expression groups by *k*-means clustering of the sum of their pathprint scores in the common self-renewal pathways. High scores were associated with poor prognosis in each of the studies, and were also significant compared with a background of random pathway permutations (Figure 4b; see Additional file 11). The identification of translation factors suggests that modulation of translation might be a therapeutic approach in poor-prognosis AML, consistent with studies targeting this process in early-phase clinical trials [54]. The set of stem cell pathways that are conserved across human and mouse have significantly greater clinical relevance than do either the human or mouse pathways on their own, demonstrating the value of a cross-species analysis in this case study (see Additional file 12). The GPCR, PLCG2, and RAN modules may represent new pathways for clinical investigation; a clear relationship between the pathprint score and clinical outcome was found for the PLCG2 module, which comprises a tightly connected set of genes involved in signaling and metabolism (Figure 4c; see Additional file 13).

## Conclusions

The pathprinting project provides the scientific community with a consistent, functional annotation of gene expression across a fixed 'set' of pathways. It moves beyond traditional approaches, resolving the major bottleneck on the road towards efficient systems biology-based modeling by addressing the inherent experimental and platform biases that confound microarray analyses. Pathprinting is now being applied to group the function of datasets within the Harvard Stem Cell Institute Stem Cell Commons [55] so that samples that have similar function can be discovered within stem cell data. A Cytoscape plug-in is also in development as part of the National Heart, Lung, and Blood Institute Progenitor consortium [56], and we have integrated the method into the Stem Cell Discovery Engine (SCDE) [57] to provide web-based accessibility. The SCDE is a portal for integrated access to tissue and cancer stem cell experimental information and molecular profiling analysis tools via a web-based Galaxy instance. Pathprinting is also embedded within the toolbench distribution of Galaxy. We encourage the community to employ pathprinting to communicate functional findings more consistently. It is important to note that pathprinting is effective for use on single samples; a sample can easily be pathprinted and compared with 'what is there'. This has important implications for applications in personalized medicine and single cell analyses.

The R package Pathprint is provided to calculate pathprints (or continuous pathway scores) from expression arrays and pathway enrichments from input gene lists. The package also contains a database of approximately 180,000 pathprints from GEO. The packages, along with Sweave files detailing the package usage and analysis in this paper are available online [43]. A supplementary package, pathprintTF, is also provided, containing a similar framework and database to pathprint but built upon protein interaction modules centered on transcription factors rather than pathways to enable cross-platform comparison of transcriptional control elements. The transcription factor modules are based on protein-protein interaction sub-networks centered on a series of 1,022 transcription factors. The package and more details are provided on the Pathprint website.

The correlation of mRNA expression with protein levels, and also with phenotype, depends on a variety of factors such as translation efficiency, mRNA abundance, ribosome occupancy, and protein abundance and turnover. Gene expression levels are a good surrogate for protein levels for housekeeping genes (ribosomal proteins, glycolytic enzymes, and tricarboxylic acid cycle proteins) but mRNA levels correlate less well with protein levels for kinases, proteases, secreted proteins and transcription factors, and overall mRNA variability explains only approximately 40% of the variability in protein levels. Pathprinting establishes a standardized method for large-scale quantitative comparisons of cellular function, and any analysis of this type depends on the availability of large-scale quantitative genome-wide datasets. Gene expression data repositories are currently the only resource expansive enough to address this need. Future versions of Pathprint will extend the value of existing array data by integrating RNA-sequencing, epigenetic and proteomic profiles, providing context for new experiments from the existing body of microarray data, and helping resolve the links between regulation and expression of cellular function.

## Abbreviations

AML: acute myeloid leukemia; ESC: Embryonic stem cell; GEB: Gene Expression Barcode GEO: Gene Expression Omnibus; GSEA: Gene Set Enrichment Analysis; GO: Gene Ontology; GPCR: G protein-coupled receptor; KEGG: Kyoto Encyclopedia of Genes and Genomes; ID: identification; iPSC: Induced pluripotent stem cell; PLCG2: 1-phosphatidylinositol-4,5-bisphosphate phosphodiesterase gamma-2; POE: Probability of expression; RAN: RAS-related nuclear protein; SCDE: Stem Cell Discovery Engine; SD: standard deviation; SRAS: Self renewal-associated signature.

## Competing interests

The authors declare that they have no competing interests.

## Authors' contributions

GA, OH, and WH conceived the study; GA designed the algorithm, analyzed the data, and prepared the manuscript; WH supervised the project; WH and OH edited the manuscript; IK and LS constructed the static networks; RP and TC designed the benchmarking; AK and SA designed the biological validations; GA and US compiled the R package; and SH integrated the package into SCDE. All authors read and approved the final manuscript.

## Supplementary Material

Additional file 1 **Table listing platforms covered by Pathprint**. Click here for file

Additional file 2 **Table listing pathway sources, retrieval dates, website addresses, and the top Gene Ontology (GO) term that is enriched in each pathway (hypergeometric distribution *P*-value)**. Click here for file

Additional data 3 **Table of the overlap in the genes covered by each gene set resource across in Pathprint (human pathways)**. Click here for file

Additional file 4 **Supplementary Figure 1. Benchmarking and threshold optimization**. Benchmarking is based on the tissue dataset (above) and brain sub-types (below). **(a,d) **Mean error rate based on ten repeats of a five-fold cross-validation over a range of probability of expression (POE) thresholds. Error bars indicate -/+ 1SD. The black line indicates the ratio for the unthresholded POE matrix, and the red for the Gene Expression Barcode (GEB), and dashed lines indicate -/+ 1SD. **(b,d) **Intra-cluster versus inter-cluster variance ratio over a range of POE thresholds. Dashed line indicates the ratio for the unthresholded POE matrix. **(c,f) **Mean average precision over a range of POE thresholds for the pathprint (black circles) and a pathprint build on random gene sets of equivalent size distribution (blue circles). Solid lines indicate the mean average precision for GEB (blue), Spearman correlation (green), and the unthresholded pathprint (red). NB: GEB or gene expression correlation data were not calculated for the brain subtype dataset. Click here for file

Additional file 5 **Supplementary Figure 2. Precision-recall curves across the full set of Gene Expression Omnibus (GEO) samples and distribution of distances of GEO samples from each tissue pathprint: (a) **Precision-recall curves for each of the tissues across the pathprint-mapped GEO database; brain (red), kidney (yellow), liver (green), lung (cyan), skeletal muscle (blue), and spleen (magenta). **(b) **Precision curves for each of the tissues across the pathprint-mapped GEO database (red; right axis) and histogram of distance of samples in the pathprint-mapped GEO database from each tissue consensus pathprint (black; left axis). Distance scales between 0 (all pathway scores matched) to 1 (all pathway scores mismatched, that is, 1 versus -1). **(c) **Estimated *P*-values: A *P*-value was assigned to every sample in the GEO pathprint matrix to assess the likelihood of association with the consensus pathprint for each tissue. The plots the relationship between this *P*-value and the precision (that is, the proportion correctly matched to each tissue), as determined from the GEO metadata, when samples are ranked according to *P*-value. Click here for file

Additional file 6 **Table of the *Rattus norvegicus*, *Danio rerio*, *Drosophila melanogaster*, and *Caenorhabditis elegans *arrays that are most closely matched to human/mouse brain and liver samples**. Click here for file

Additional file 7 **Table listing the pathways in the pluripotent consensus pathprint**. Click here for file

Additional file 8 **Supplementary Figure 3. Embryonic stem cell (ESC) differentiation timecourse**. **(a) **Distance from the ESC pathprint signature of two mouse ESC lines, J1 and R1, differentiating to embryoid bodies. The data were obtained from Gene Expression Omnibus (GEO) accessions GSE2972 (J1) and GSE3749 (R1). **(b) **Heat-map of pathways in the ESC pathprint signature that varied over both differentiation time courses (blue = -1, white = 0, red = +1). The column labeled 'ES' denotes the ESC pathprint signature. Click here for file

Additional file 9 **Table listing the pluripotent seed arrays and top arrays matching the pluripotent consensus pathprint**. Click here for file

Additional file 10 **Supplementary Figure 4. Combined human and mouse blood lineage tree**: Pathway heat-map based on shared informative pathways that resolve trees (b) and (c) in Figure 2. Click here for file

Additional file 11 **Supplementary Figure 5. Pathway-based survival analysis**. **(a) **Kaplan-Meier curves of patients in four independent acute myeloid leukemia (AML) clinical datasets stratified by expression of common mouse and human self renewal-associated signature (SRAS) pathways; translation factors (Wikipathways), G protein-coupled receptors (GPCRs), class B secretin-like (Wikipathways), 1-phosphatidylinositol-4,5-bisphosphate phosphodiesterase gamma-2 (PLCG2) (static module), and RAS-related nuclear protein (RAN) (static module). The red and blue lines indicate high and low pathprint scores respectively **(b) ***P*-value of Kaplan-Meier estimate of patients stratified by expression of common mouse and human SRAS pathways in four independent clinical datasets, relative to a background of randomly selected pathways from the full pathprint set, **(c) **Common genes relative to a background of randomly selected genes from expression chip (only single dataset shown), and **(d) **common SRAS pathways relative to a background of randomly selected human SRAS pathways. A red dot indicates the *P*-value; the grey cone is a bean plot representing the distribution of *P*-values from 1,000 randomly selected sets of pathways or genes; and the blue line indicates *P *= 0.05. Click here for file

Additional file 12 **Supplementary Figure 6. Pathway-based survival analysis by species**. Kaplan-Meier curves of patients with acute myeloid leukemia (AML) stratified by expression of **(a) **common human and mouse, **(b) **human, and **(c) **mouse self-renewal-associated signature (SRAS) pathways in four independent clinical datasets. The red and blue lines indicate high and low pathprint scores, respectively. Click here for file

Additional file 13 **Supplementary Figure 7. The 1-phosphatidylinositol-4,5-bisphosphate phosphodiesterase gamma-2 (PGLC2) module**. **(a) **The protein-protein interaction network of a single human/mouse common self renewal-associated signature (SRAS) pathway: the PGLC2 module. Node color represents fold change in the combined leukemic/normal blood dataset (expression in normal and leukemia stem cells divided by expression in progenitor cells). **(b) **The pathprint score of this single pathway in patients with acute myeloid leukemia (AML) was associated with survival in four independent clinical datasets (red, +1; yellow, 0; blue, -1) Click here for file
