# Supplementary figures and images for: Pathprinting: An integrative approach to understand the functional basis of disease
Source: Genome Med. 2013 Jul 26;5(7):68. doi: 10.1186/gm472 (PMC3971351; doi:10.1186/gm472)

a)

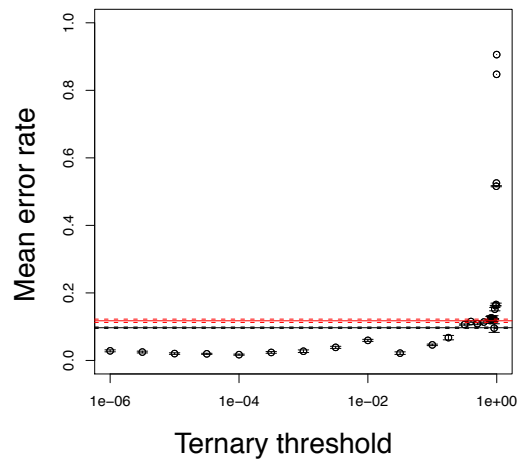

b)

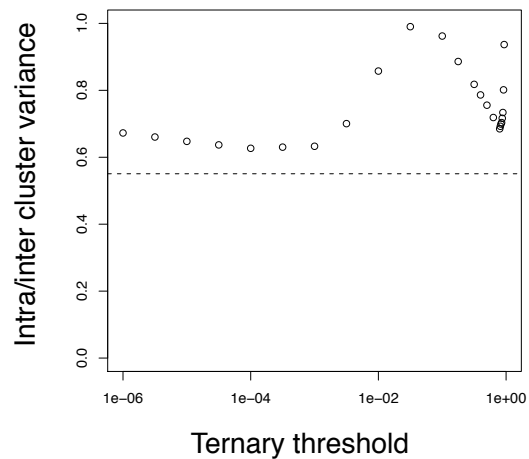

c)

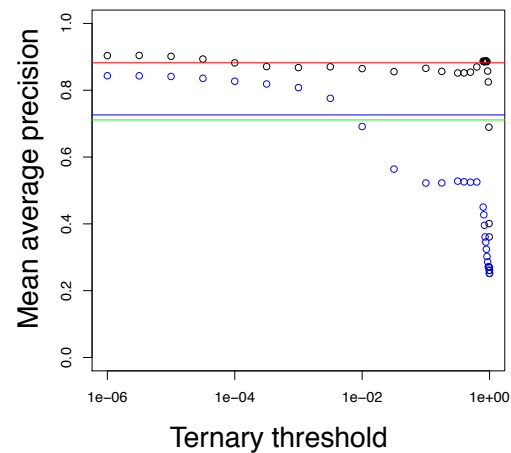

d)

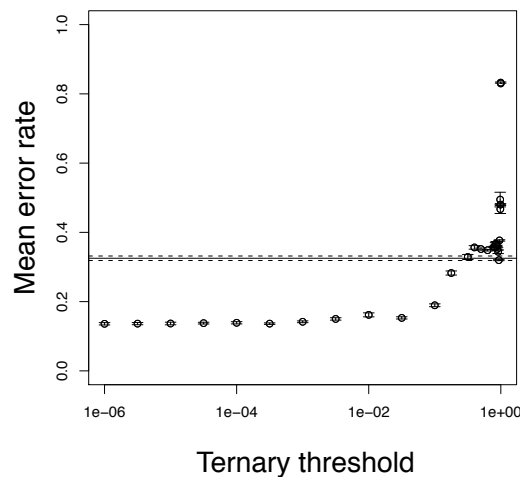

e)

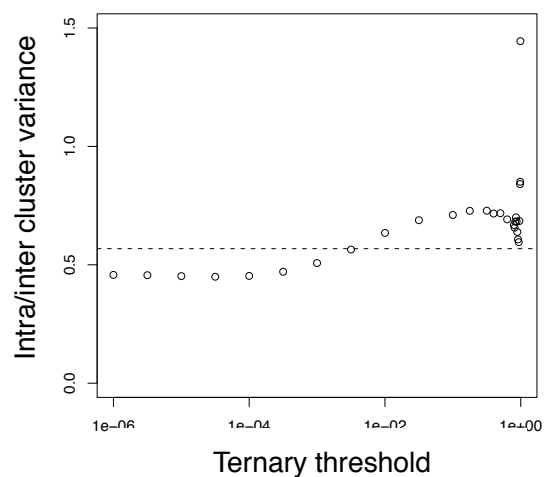

f)

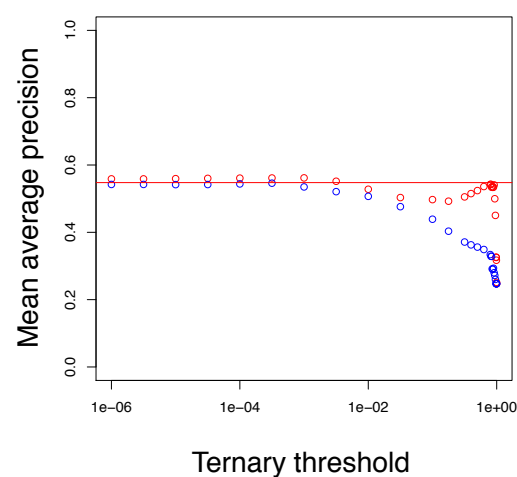

Supplement: Additional file 4 — Supplementary Figure 1. Benchmarking and threshold optimization. Benchmarking is based on the tissue dataset (above) and brain sub-types (below). (a,d) Mean error rate based on ten repeats of a five-fold cross-validation over a range of probability of expression (POE) thresholds. Error bars indicate -/+ 1SD. The black line indicates the ratio for the unthresholded POE matrix, and the red for the Gene Expression Barcode (GEB), and dashed lines indicate -/+ 1SD. (b,d) Intra-cluster versus inter-cluster variance ratio over a range of POE thresholds. Dashed line indicates the ratio for the unthresholded POE matrix. (c,f) Mean average precision over a range of POE thresholds for the pathprint (black circles) and a pathprint build on random gene sets of equivalent size distribution (blue circles). Solid lines indicate the mean average precision for GEB (blue), Spearman correlation (green), and the unthresholded pathprint (red). NB: GEB or gene expression correlation data were not calculated for the brain subtype dataset. [file gm472-S4.PDF]

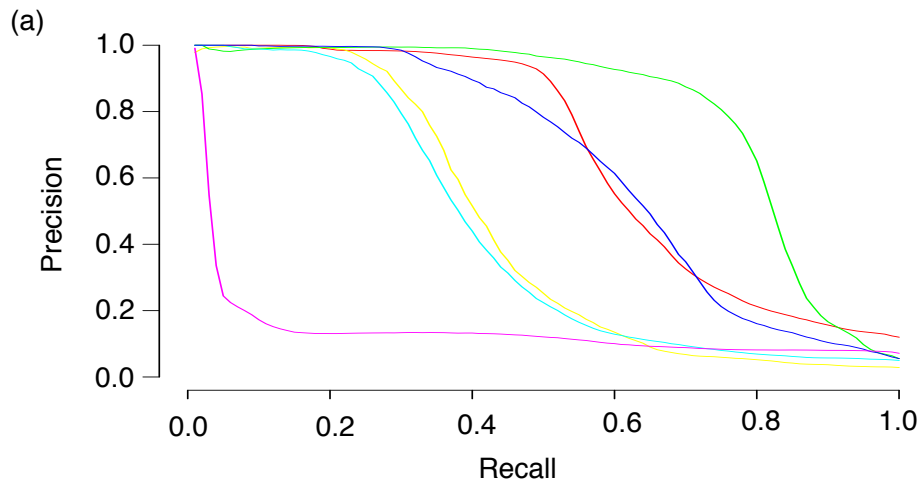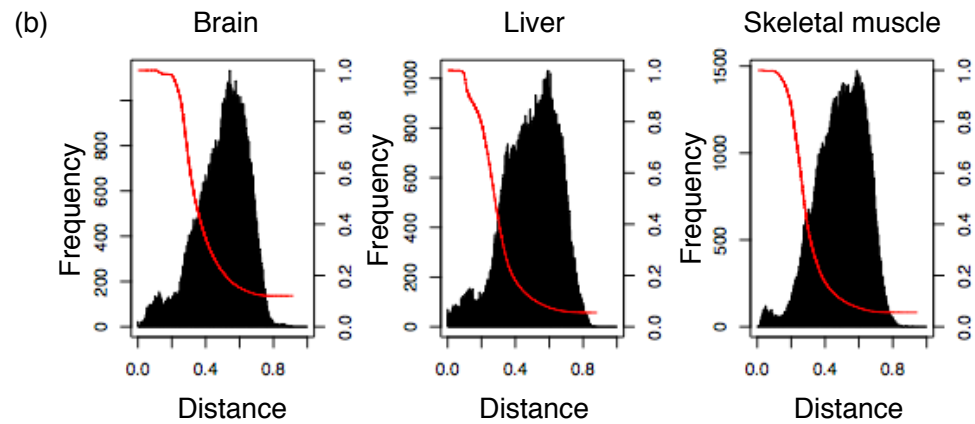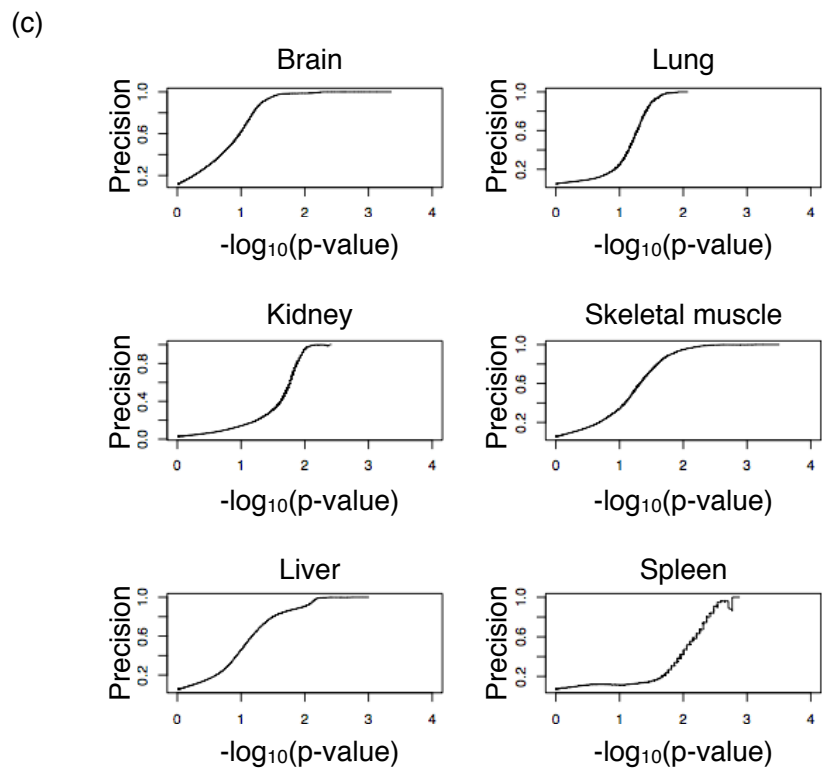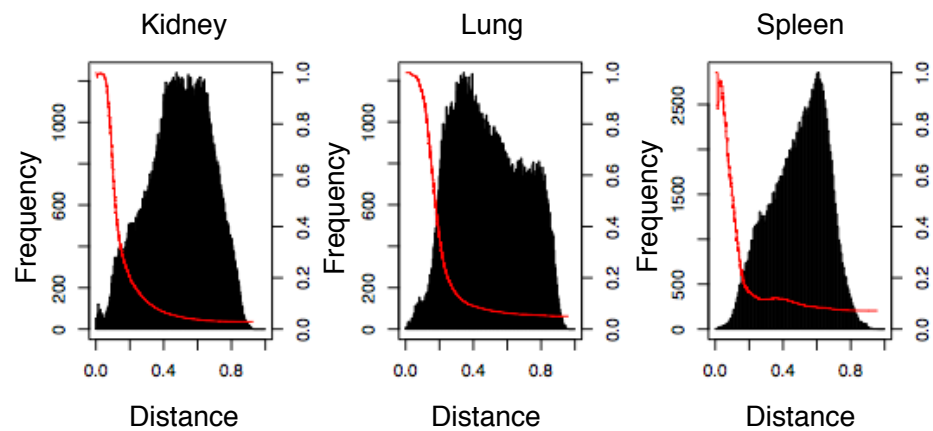

Supplement: Additional file 5 — Supplementary Figure 2. Precision-recall curves across the full set of Gene Expression Omnibus (GEO) samples and distribution of distances of GEO samples from each tissue pathprint: (a) Precision-recall curves for each of the tissues across the pathprint-mapped GEO database; brain (red), kidney (yellow), liver (green), lung (cyan), skeletal muscle (blue), and spleen (magenta). (b) Precision curves for each of the tissues across the pathprint-mapped GEO database (red; right axis) and histogram of distance of samples in the pathprint-mapped GEO database from each tissue consensus pathprint (black; left axis). Distance scales between 0 (all pathway scores matched) to 1 (all pathway scores mismatched, that is, 1 versus -1). (c) Estimated P-values: A P-value was assigned to every sample in the GEO pathprint matrix to assess the likelihood of association with the consensus pathprint for each tissue. The plots the relationship between this P-value and the precision (that is, the proportion correctly matched to each tissue), as determined from the GEO metadata, when samples are ranked according to P-value. [file gm472-S5.PDF]

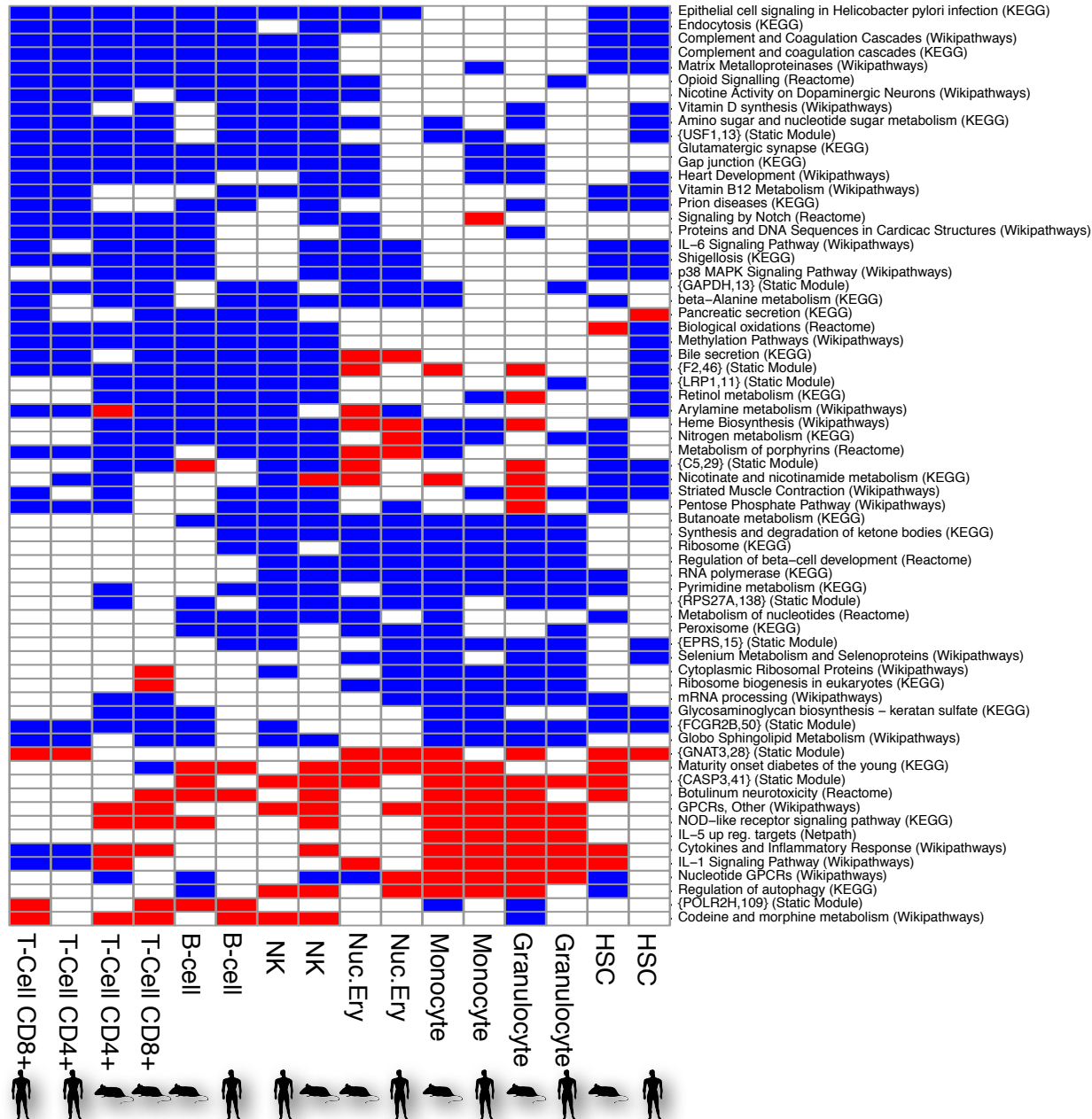

Supplement: Additional file 10 — Supplementary Figure 4. Combined human and mouse blood lineage tree: Pathway heat-map based on shared informative pathways that resolve trees (b) and (c) in Figure 2. [file gm472-S10.PDF]

(a)

GSE10358,  $p < 0.008$ 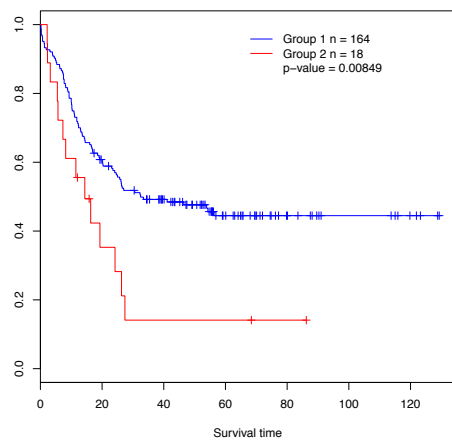GSE12417,  $p < 0.086$ 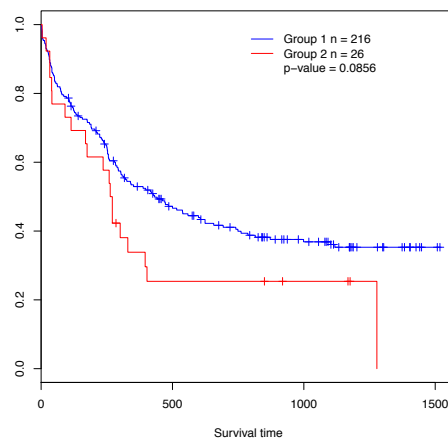GSE1159,  $p < 0.0009$ 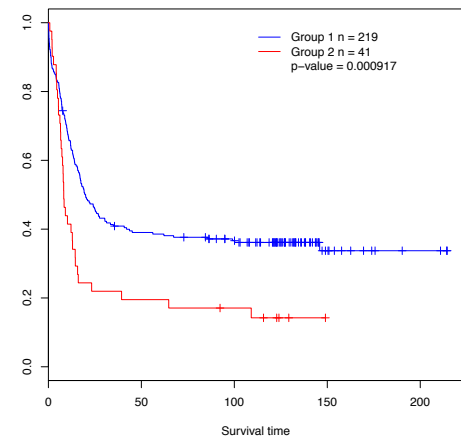GSE14468,  $p < 0.008$ 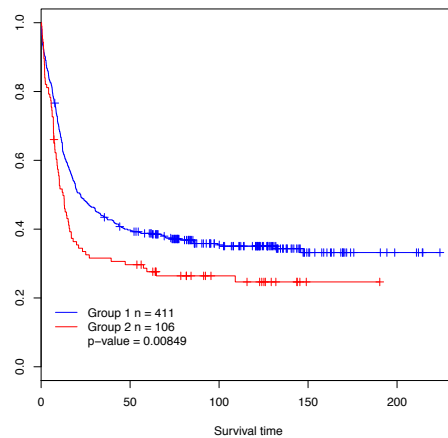

(b)

GSE10358,  $p < 0.008$ 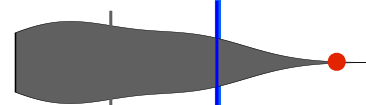GSE12417,  $p < 0.086$ 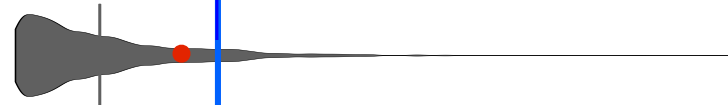GSE1159,  $p < 0.0009$ 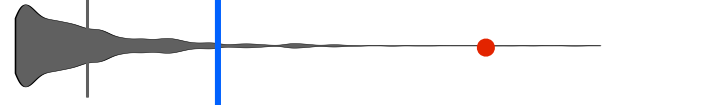GSE14468,  $p < 0.008$ 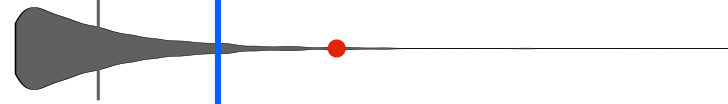

(c)

GSE10358,  $p < 0.042$ 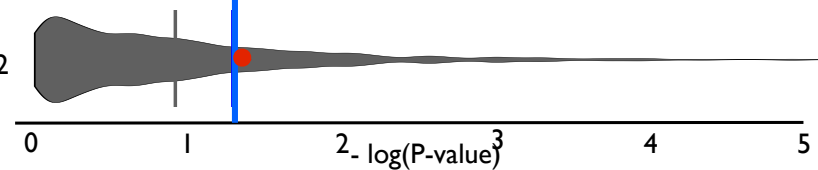

(d)

GSE10358,  $p < 0.008$ 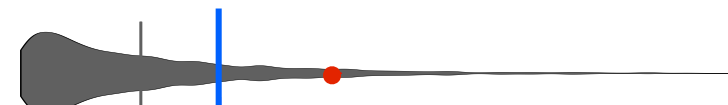GSE12417,  $p < 0.086$ 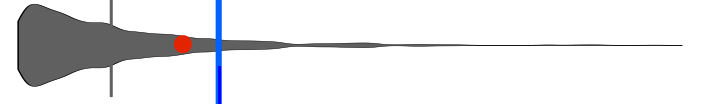GSE1159,  $p < 0.0009$ 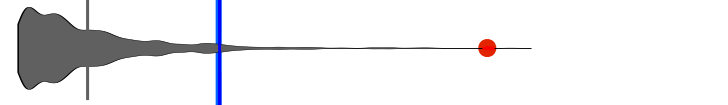GSE14468,  $p < 0.008$ 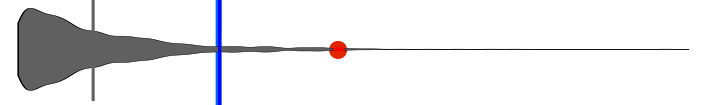

Supplement: Additional file 11 — Supplementary Figure 5. Pathway-based survival analysis. (a) Kaplan-Meier curves of patients in four independent acute myeloid leukemia (AML) clinical datasets stratified by expression of common mouse and human self renewal-associated signature (SRAS) pathways; translation factors (Wikipathways), G protein-coupled receptors (GPCRs), class B secretin-like (Wikipathways), 1-phosphatidylinositol-4,5-bisphosphate phosphodiesterase gamma-2 (PLCG2) (static module), and RAS-related nuclear protein (RAN) (static module). The red and blue lines indicate high and low pathprint scores respectively (b) P-value of Kaplan-Meier estimate of patients stratified by expression of common mouse and human SRAS pathways in four independent clinical datasets, relative to a background of randomly selected pathways from the full pathprint set, (c) Common genes relative to a background of randomly selected genes from expression chip (only single dataset shown), and (d) common SRAS pathways relative to a background of randomly selected human SRAS pathways. A red dot indicates the P-value; the grey cone is a bean plot representing the distribution of P-values from 1,000 randomly selected sets of pathways or genes; and the blue line indicates P = 0.05. [file gm472-S11.PDF]

(a)

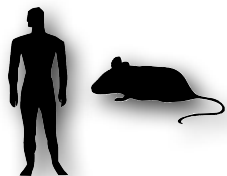

GSE14468

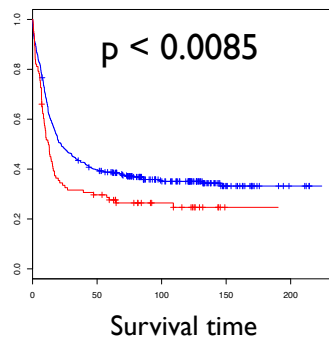

GSE12417

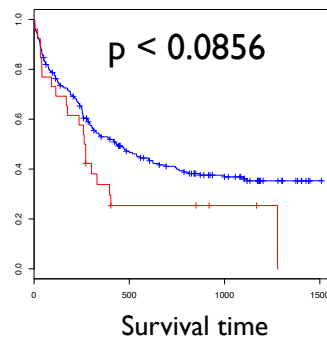

GSE10358

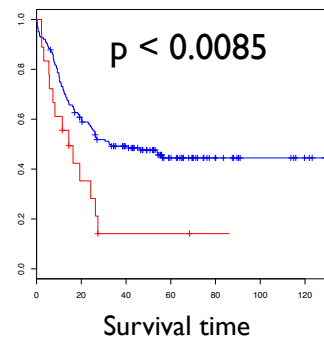

GSE1159

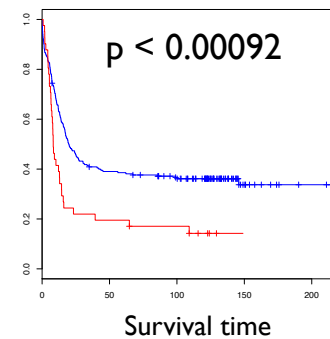

(b)

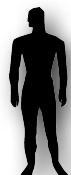

$p < 0.953$

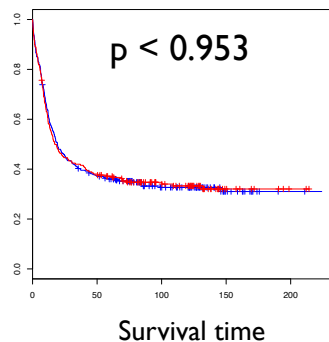

$p < 0.616$

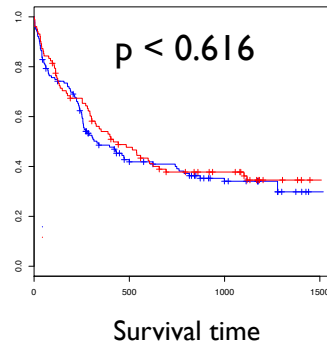

$p < 0.638$

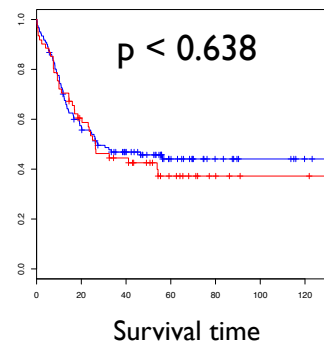

$p < 0.934$

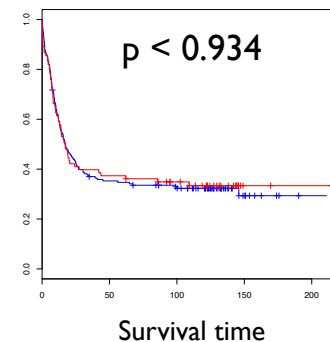

(c)

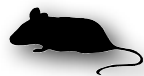

$p < 0.153$

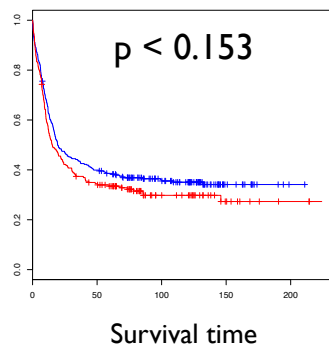

$p < 0.749$

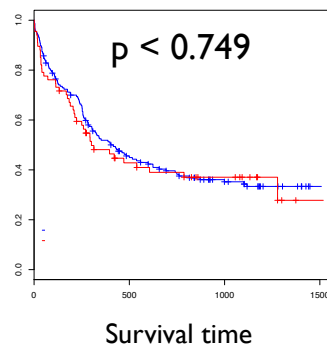

$p < 0.0105$

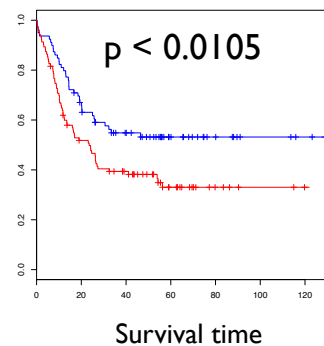

$p < 0.54$

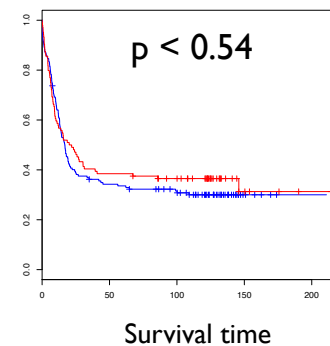

Supplement: Additional file 12 — Supplementary Figure 6. Pathway-based survival analysis by species. Kaplan-Meier curves of patients with acute myeloid leukemia (AML) stratified by expression of (a) common human and mouse, (b) human, and (c) mouse self-renewal-associated signature (SRAS) pathways in four independent clinical datasets. The red and blue lines indicate high and low pathprint scores, respectively. [file gm472-S12.PDF]

a)

Log<sub>2</sub> fold change  
(stem / progenitor)

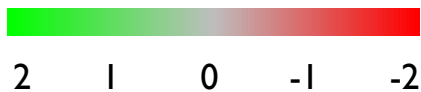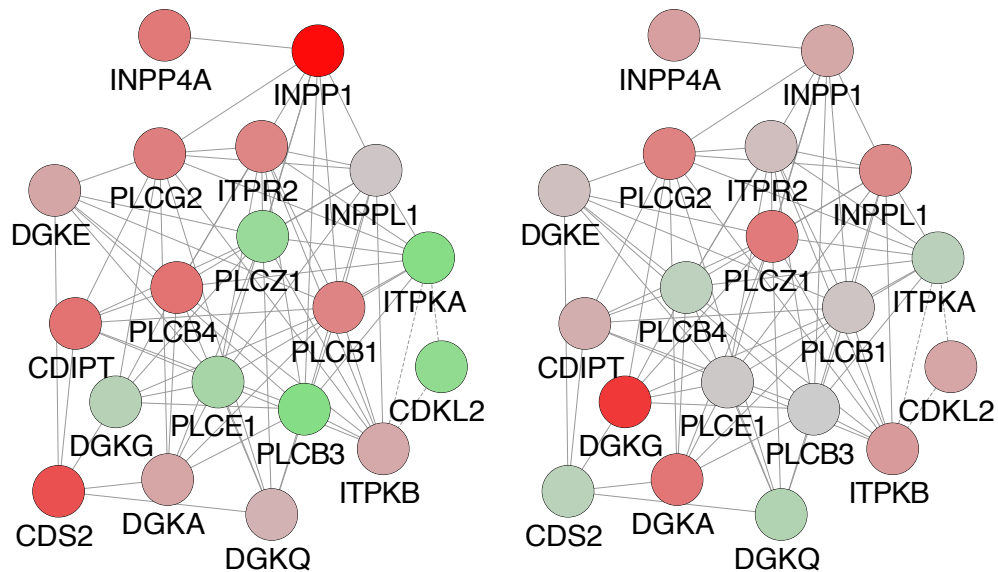

b)

GSE10358

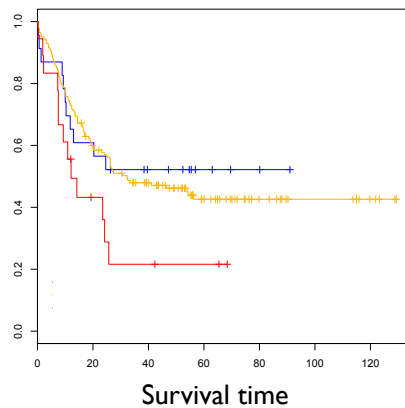

GSE12417

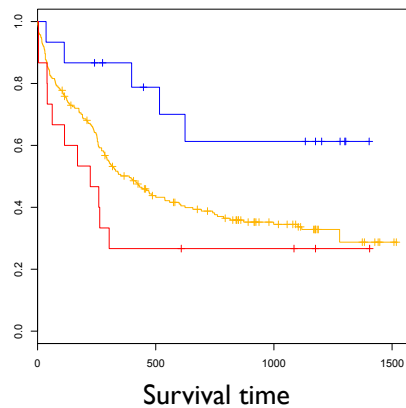

GSE1159

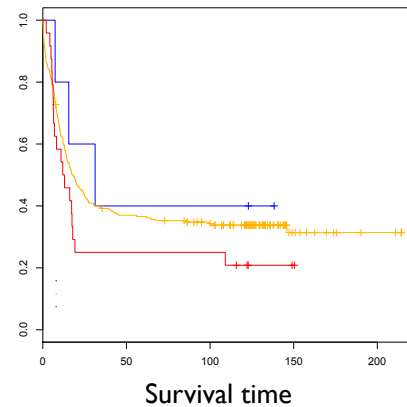

GSE14468

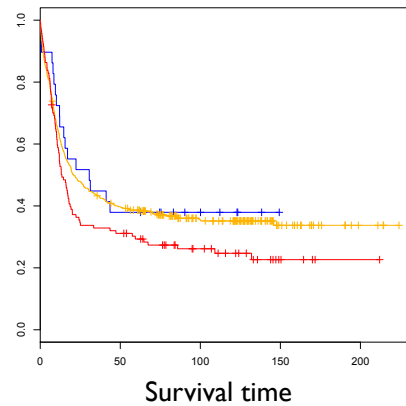

Supplement: Additional file 13 — Supplementary Figure 7. The 1-phosphatidylinositol-4,5-bisphosphate phosphodiesterase gamma-2 (PGLC2) module. (a) The protein-protein interaction network of a single human/mouse common self renewal-associated signature (SRAS) pathway: the PGLC2 module. Node color represents fold change in the combined leukemic/normal blood dataset (expression in normal and leukemia stem cells divided by expression in progenitor cells). (b) The pathprint score of this single pathway in patients with acute myeloid leukemia (AML) was associated with survival in four independent clinical datasets (red, +1; yellow, 0; blue, -1) [file gm472-S13.PDF]
