# Supplementary material for: Pathprinting: An integrative approach to understand the functional basis of disease
Source: Genome Med. 2013 Jul 26;5(7):68. doi: 10.1186/gm472 (PMC3971351; doi:10.1186/gm472)
Supplement: Additional file 8 — Supplementary Figure 3. Embryonic stem cell (ESC) differentiation timecourse. (a) Distance from the ESC pathprint signature of two mouse ESC lines, J1 and R1, differentiating to embryoid bodies. The data were obtained from Gene Expression Omnibus (GEO) accessions GSE2972 (J1) and GSE3749 (R1). (b) Heat-map of pathways in the ESC pathprint signature that varied over both differentiation time courses (blue = -1, white = 0, red = +1). The column labeled 'ES' denotes the ESC pathprint signature. [file gm472-S8.PDF]

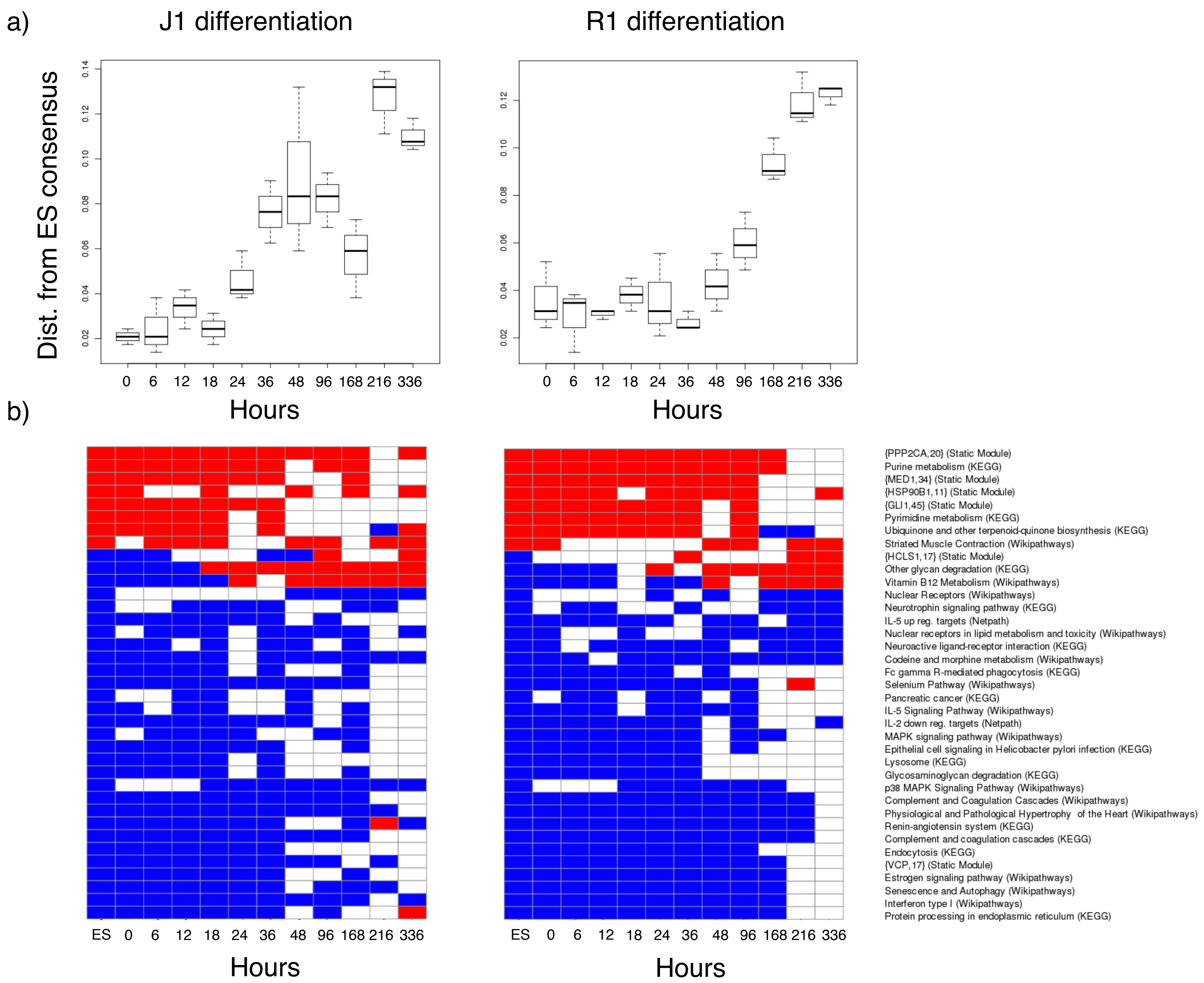
a)
J1 differentiation
R1 differentiation
Dist. from ES consensus
0.02
0.04
0.06
0.08
0.10
0.12
0.14
0 6 12 18 24 36 48 96 168 216 336
Hours
b)
ES 0 6 12 18 24 36 48 96 168 216 336
Hours
{PPP2CA,20} (Static Module)
Purine metabolism (KEGG)
{MED1,34} (Static Module)
{HSP90B1,11} (Static Module)
{GLI1,45} (Static Module)
Pyrimidine metabolism (KEGG)
Ubiquinone and other terpenoid-quinone biosynthesis (KEGG)
Striated Muscle Contraction (Wikipathways)
{HCLS1,17} (Static Module)
Other glycan degradation (KEGG)
Vitamin B12 Metabolism (Wikipathways)
Nuclear Receptors (Wikipathways)
Neurotrophin signaling pathway (KEGG)
IL-5 up reg. targets (Netpath)
Nuclear receptors in lipid metabolism and toxicity (Wikipathways)
Neuroactive ligand-receptor interaction (KEGG)
Codeine and morphine metabolism (Wikipathways)
Fc gamma R-mediated phagocytosis (KEGG)
Selenium Pathway (Wikipathways)
Pancreatic cancer (KEGG)
IL-5 Signaling Pathway (Wikipathways)
IL-2 down reg. targets (Netpath)
MAPK signaling pathway (Wikipathways)
Epithelial cell signaling in Helicobacter pylori infection (KEGG)
Lysosome (KEGG)
Glycosaminoglycan degradation (KEGG)
p38 MAPK Signaling Pathway (Wikipathways)
Complement and Coagulation Cascades (Wikipathways)
Physiological and Pathological Hypertrophy of the Heart (Wikipathways)
Renin-angiotensin system (KEGG)
Complement and coagulation cascades (KEGG)
Endocytosis (KEGG)
{VCP,17} (Static Module)
Estrogen signaling pathway (Wikipathways)
Senescence and Autophagy (Wikipathways)
Interferon type I (Wikipathways)
Protein processing in endoplasmic reticulum (KEGG)
